# Transcriptome analysis of cyst formation in *Rhodospirillum centenum* reveals large global changes in expression during cyst development

**DOI:** 10.1186/s12864-015-1250-9

**Published:** 2015-02-13

**Authors:** Qian Dong, Carl E Bauer

**Affiliations:** Molecular and Cellular Biochemistry Department, Indiana University, Bloomington, IN 47405 USA

**Keywords:** Photosynthetic *Azospirillum*, Encystment, Desiccation resistance

## Abstract

**Background:**

*Rhodospirillum centenum* is a photosynthetic member of the Gram-negative *Azospirillum* clade members of which exhibit a complex developmental life-cycle featuring morphologically distinct cell types. Under periods of nutrient deprivation, replicative vegetative cells differentiate into metabolically dormant cysts that survive harsh environmental stresses such as desiccation. Encystment involves a multi-stage developmental process that includes the rounding of cells, production of large intracellular storage granules of poly-hydroxybutyrate (PHB) and the excretion of a protective exopolysaccharide coating that envelops dormant cysts.

**Results:**

To study the process of cyst development, we performed RNA-seq studies on cells that were induced to undergo cyst development. To assay for temporal changes in gene expression, RNA was extracted at 4, 24, 48, 72, 96 hours during development and subjected to deep sequence analysis. These results show that 812 genes exhibit log_2_ ≥ 1.5-fold changes in expression over a 96 hour cyst induction period demonstrating large global changes in gene expression during cyst development.

**Conclusions:**

Notable changes in expression occurred in numerous genes involved in cell wall and lipid biosynthesis, metabolic enzymes, and numerous regulatory genes such as histidine kinases and transcription factors. Many genes involved in protein synthesis and DNA replication were also significantly reduced during late stages of cyst development. Genes previously identified by genetic screens as being critical for cyst development also exhibited changes of expression during cyst induction. This study provides the first transcriptome profile of global changes in gene expression that occur during development of cysts in a Gram-negative species.

**Electronic supplementary material:**

The online version of this article (doi:10.1186/s12864-015-1250-9) contains supplementary material, which is available to authorized users.

## Background

Many bacteria survive unfavorable conditions by differentiating into metabolically quiescent resting stages, commonly referred to as cysts or spores [1]. Production of resting cells involves major rearrangements of physiology, ultrastructure and biochemical composition [2-4]. Resting stage differentiation in prokaryotes is diverse and in some model systems is known to be regulated by complex and hierarchical signal transduction pathways [4].

The formation of endospores has been intensively investigated in Gram-positive species such as *Bacillus subtilis,* which forms endospores in response to unfavorable growth conditions [4,5]. Gram-positive spores display exceptional resistance to physical and chemical stresses such as desiccation, high temperatures (>100°C), radiation, oxidizing agents and pressure [3,6,7]. These spores arise from asymmetric cell division, which produces a prespore that becomes engulfed, matured, and then released by lysis of the mother cell. Less characterized are resting cysts produced by Gram-negative bacteria. Resting cysts produced by this group do not survive high temperatures and pressure but do provide resistance to desiccation and to moderate heat stress. The best-studied Gram-negative resting cells are myxospores synthesized by myxobacteria, a group of soil inhabiting delta proteobacteria. Myxospores from *Myxococcus xanthus* are produced by transforming an entire vegetative cell into a dormant cell. As is typical of cysts produced by other Gram-negative genera, myxospores are only moderately resistant to heat (up to 60°C), as well as resistant to desiccation, sonication, UV-irradiation, detergents and enzymatic digestion [8].

In addition to myxobacteria, there are agronomically important soil-inhabiting members of the genera *Azospirillum* and *Azotobacter* that produce desiccation and moderate heat resistant cysts [9,10]. Members of the *Azospirillum* clade are associated with root rhizospheres in a broad range of plants [11]. These aerobic nitrogen fixing organisms are capable of stimulating plant growth through the donation of both bacterially synthesized fixed nitrogen and plant hormones such as indole-3-acetic, gibberellins and cytokinins [12]. Inoculating fields and/or seeds with *Azospirillum* sp. are known to significantly enhance crop yields for a wide diversity of cultivars including corn and wheat [13]. *Azospirillum* encystment involves morphological transitions during which cells round up to form a thick outer exopolysaccharide coat termed the exine layer [14]. The formation of cysts also correlates with the appearance of intracellular poly-hydroxybutyrate (PHB) granules that are presumably used as energy reserves [15,16]. Once water and nutrients are available, cysts germinate by reforming vegetative cells that emerge from the exine coat. Beyond myxobacteria, *Azospirillum* and *Azotobacter* there are several Gram-negative pathogens such as *Legionella pneumophila* that also produce dormant cysts [17,18]. However, little is known about the biology of cyst development in pathogenic species.

Analysis of the regulation of resting cell synthesis has been extensively studied in Gram-positive organisms. In characterized model systems such as *B. subtilis,* the induction of endospore development is tightly regulated by a complex of well-studied hierarchical signal transduction pathways [19,20]. However, among Gram-negative organisms, there is much less known about the control of resting cell development. The best-characterized system is *M. xanthus,* however many molecular details on the control of myxospores development remain lacking. To further expand the understanding of resting cell development among Gram-negative organisms, we have undertaken detailed genetic and biochemical studies of cyst formation by *Rhodospirillum centenum,* a photosynthetic member of the *Azospirillum* clade. These studies have identified numerous regulatory factors that contribute to the control of encystment which include the involvement of five histidine kinases and a CtrA transcription regulator [21-26]. Cyst production is also influenced by production of cGMP and subsequent binding of cGMP by a CRP homolog [27]. We have also shown that the timing of cyst development is regulated by a Che-like signal transduction cascade that contains a methyl-accepting chemotaxis receptor and several chemotaxis-like regulators [22,23,26]. Null mutations of these effectors fall within phenotypes that either block or activate encystment.

To further an understanding of how a plethora of regulatory proteins control cyst development, we must first have an understanding of what global changes in transcription occur as cells transition from vegetative growth into the cyst developmental pathway. Once baseline changes in expression are established, we can then address how individual regulatory mutations affect these global changes in expression. Towards this goal, we have utilized RNA-seq based high-throughput DNA sequencing technology to profile and quantify gene expression changes that occur in *R. centenum* as they develop into cysts. Up to now, there is only one example of global transcriptome analysis of resting cell development in a Gram-negative organism. Specifically, a DNA chip array study was used to provide an analysis of large changes in gene expression that occur during myxospore development [28]. However, there is no report of transcriptome profiling of a Gram-negative organism undergoing the cyst development pathway using high resolution RNA–seq analysis. Studies have established RNA-seq as a powerful tool for transcriptional analysis as it provides unprecedented global resolution and depth of transcription profiles [29-31]. In this report, we have performed RNA-seq analysis of *R. centenum* as it undergoes cyst development over a multi-step time course. This high-resolution analysis provides the first detailed understanding of global changes in expression that occur at different stages of Gram-negative cyst development. The results of this study not only show alterations in many metabolic pathways but also alterations in numerous signal transduction regulatory networks and transcription factors such as sigma subunits. These results provide an understanding of physiological changes that occur during cyst development and additionally provide a road map for the study of similar developmental processes that occur in other cyst forming Gram-negative bacteria.

## Results

### Overview of the transcriptional profile and identification of differentially expressed genes by RNA-Seq

**Figure 1:**
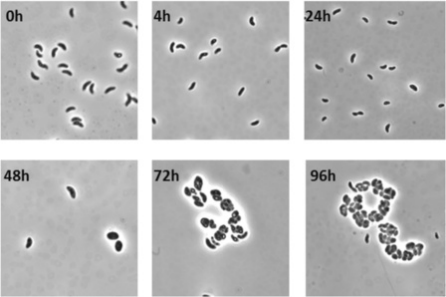
**Phase-contrast microscopy depicting representative stages in** ***R. centenum*** **cyst formation.** The panels labeled with black numbers show demonstrative cell types observed at 4 h, 24 h, 48 h, 72 h and 96 h time points after induction of cyst formation by a shift from CENS (0 h) to CENBA medium.

As previously reported, *R. centenum* undergoes induction of cyst development when shifted from a complex nutrient rich growth medium (CENS) to a nutrient poor defined medium that uses butyrate as a sole carbon source (CENBA) [16]. In this study, we performed the rich to minimal nutrient shift and monitored cell morphology during a 4-day period during which cells underwent changes in morphology along a previously observed timeline (Figure 1). At five defined time points (4, 24, 48, 72, 96 hours), samples from biological replicates were taken, chilled on ice, centrifuged and stored as pellets at -80°C. Once independent replicate sets of samples were obtained, RNA was then extracted for RNA-Seq analysis. Briefly, extracted total RNA was depleted of rRNA and then converted into a cDNA library as described in Methods. The cDNA library was then analyzed for coverage by deep sequencing using a Hiseq2000 sequencing platform. In total, over 120 million (M) (strand specific) RNA-Seq reads were obtained. 98 M of these reads were mapped on the genome of *R centenum*. This data set resulted in a single nucleotide resolution transcriptome with a sum of at least 150x coverage per nucleotide.

To avoid metabolic gene expression changes that would occur as a result of a simple nutrient downshift, we analyzed pair-wise comparisons between RNA extracted from CENBA at 4 hours with RNA extracted from CENBA at 24 hr, 48 hr, 72 hr and 96 hr. Genes were designated as differentially expressed genes (DEGs) with log_2_fold change ≥ 1.5 in at least one time point and a false-discovery-rate adjusted p-value of less than 0.05. Analysis of the resulting RNA-seq data set revealed a total of 812 DEGs, which corresponds to 19.78% of the annotated *R. centenum* genome.

**Figure 2:**
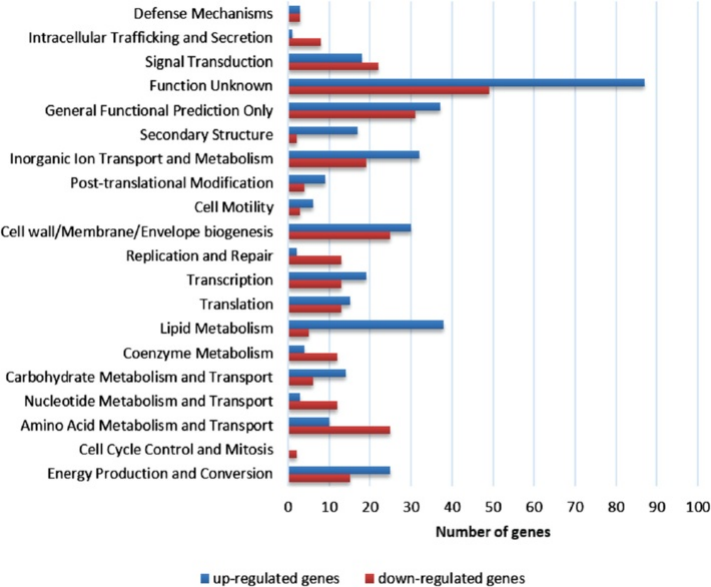
**Differentially expressed genes (DEGs) grouped by COG.** Genes were designated as DEGs with log_2_fold change ≥ 1.5 in at least one time point and a false-discovery-rate adjusted p-value of less than 0.05. RNA-seq data set revealed a total of 812 DEGs, among which a 636 genes were grouped into 20 unique COG categories. Up- and down- regulated genes are indicated by blue and red bars respectively, representing the numbers of genes per functional category.

To facilitate an overview of functional roles of individual loci we subcategorized each differentially expressed gene (DEG) into clusters of orthologous groups (COG). Among the 812 DEGs a total of 636 genes were grouped into 20 unique COG categories (Additional file 1: Table S1, Figure 2). Among these 20 subgroups, the cluster for ‘Function Unknown’ represented the largest group (136 genes), followed by ‘General Function Prediction’ (68 genes), ‘Cell wall/Membrane/Envelope biogenesis’ and ‘Inorganic Ion Transport and Metabolism’. The categories of ‘Cell Cycle Control and Mitosis’ and ‘Defense Mechanism’ represent the smallest group (Figure 2). Analysis of functional grouping details indicates that the more highly up-regulated genes tended to be in COGs categorized as Lipid Metabolism, Inorganic Ion Transport and Metabolism, Energy Production and Conversion, Transcription and Signal Transduction. The more prominent down-regulated genes tended to be in COGs categorized as Amino Acid Metabolism and Transport and Cell Wall/Membrane/Envelope biogenesis followed by Inorganic Ion Transport and Metabolism and Energy Production and Conversion (Additional file 1: Table S1). In the sections below, we discuss the identification and analysis of differentially expressed genes that are likely relevant to the encystment process in each of these 20 unique COG categories.

### Cell wall and cell membrane synthesis

**Figure 3:**
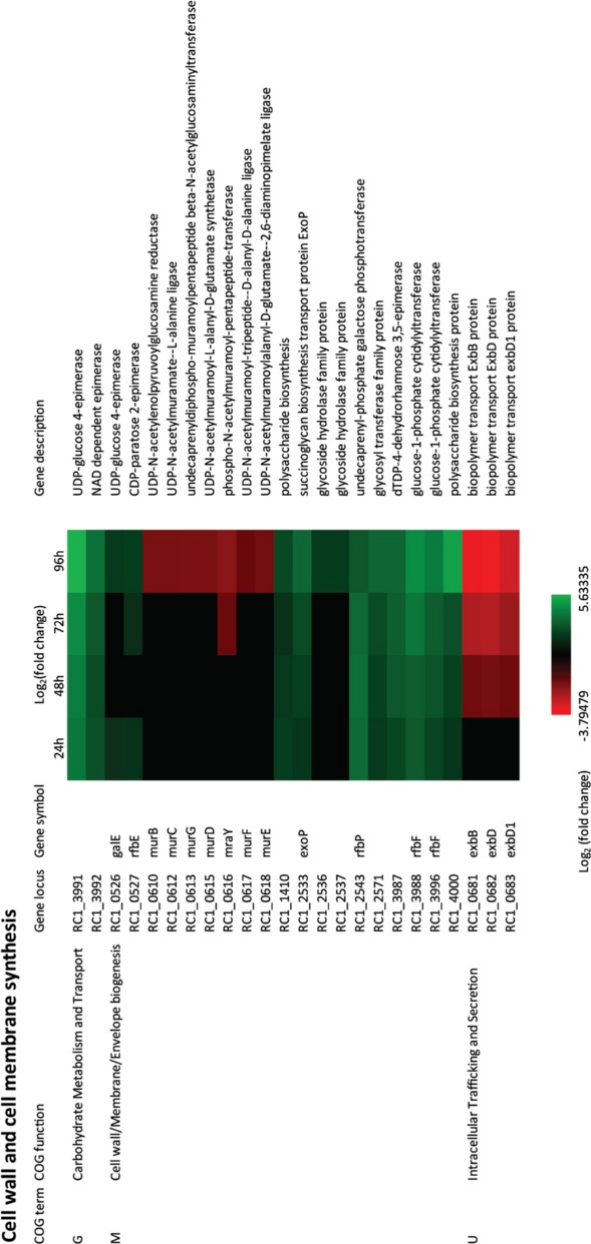
**Time course expression profiles of representative cell wall and cell membrane synthesis and transportation genes.** Each column represents sample from one time-point. The maximum relative expression is green; the minimum is red.

Microscopic observations indicate that cyst forming Gram-negative cells in the *Azospirillum* clade undergo significant alterations in cell wall morphology when transitioning from vegetative to cyst cells [15]. For example, *R. centenum* vegetative cells have typical Gram-negative cell wall morphology comprised of outer and inner membranes [32-34]. When transitioning from vegetative to cyst forms, these cells are known to eject their flagella, round up and build a thick exopolysaccharide coat called an exine layer [16]. It is therefore not surprising that four COGs containing genes involved in cell wall biogenesis have numerous up- and down-regulated changes in gene expression during cyst development. For example, COG M and COG G, which contain genes involved in cell wall biosynthesis have numerous up-regulated exopolysaccharide biosynthesis genes in 24 hours through 96 hours (Figure 3). For example, five genes coding for enzymes that add sugars to nucleotide diphosphate derivatives during early steps in exopolysaccharide (EPS) biosynthesis (*RC1_3991, RC1_3992, RC1_3987, RC1_0526*, and *RC1_0527*) are up-regulated 3-8 fold from 24 hours through 96 hours. The same is true for the expression of seven middle and later stage enzymes involved in EPS biosynthesis (*RC1_3987, RC1_3988, RC1_3996, RC1_4000, RC1_2533, RC1_2543,* and *RC1_2571*), which are also up-regulated to a similar extent (Figure 3).

The involvement of EPS biosynthesis in encystment is underscored by prior genetic studies on cyst development by our laboratory where we have isolated a number of EPS deficient mutants that are also defective in forming viable cysts [27]. Among the genes genetically identified as hypo-cyst suppressors were a mini-Tn5 disruption of *RC1_1410* that encodes a periplasmic polysaccharide export protein whose expression increases approximately 4-fold throughout cyst development; mini-Tn5 disruptions of *RC1_2536* and *RC1_2537* that code for glycoside hydrolase family proteins that are closely related to the GT1 family of glycosyltransferases which in *E. coli* are involved in polysaccharide chain synthesis. These two proteins increase expression ~4-fold late in encystment. A mini-Tn5 disruption was also obtained for *RC1_3992* that encodes NAD-dependent epimerase/dehydratase similar to *E. coli* protein WcaG, a synthetase of the polysaccharide precursor GDP-L-fucose. All of above are predicted to be involved in cyst exine layer synthesis. There is also a putative operon coding for several secretion genes in COG U that are involved in exopolysaccharide synthesis (*exbB, exbD* and *exbD1*) that are down-regulated in 48 through 96 hours indicating that some aspects of EPS biosynthesis may also be ramping down in late stages of encystment (Figure 3).

**Figure 4:**
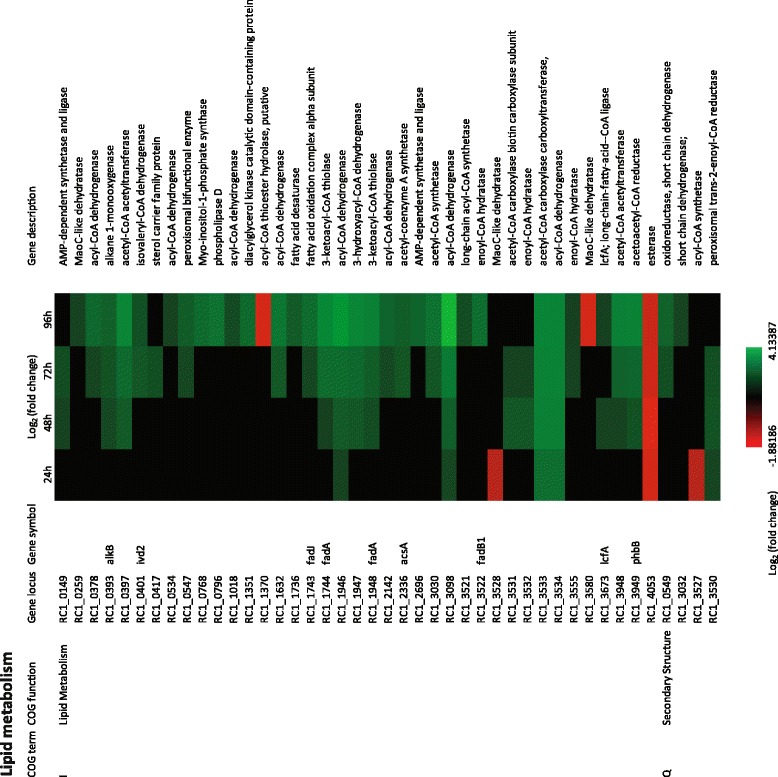
**Time course expression profiles of representative lipid metabolism and secondary structure genes.** Each column represents sample from one time-point. The maximum relative expression is green; the minimum is red.

Analysis of COG Q and COG I indicates that there are also large and significant alterations in the expression of lipid metabolism genes much of which occurs in days 3 and 4 (Figure 4). Specifically, five acyl-CoA dehydrogenases (*RC1_0378, RC1_1632, RC1_2142, RC1_3098, RC1_3534*), two acetyl CoA acetyltransferases (*RC1_0397, RC1_3948*), two fatty acid desaturases (*RC1_0393, RC1_1736*), and an inositol-1-phosphate synthase (*RC1_0768*) are all up regulated 4- to 8-fold late in cyst development (Figure 4).

When analyzing expression changes at a log_2_ 1.5 cut off there are also numerous additional cell wall and lipid metabolism genes that exhibit lower but significant 3-4 fold changes in expression (Figure 3). Among this latter group are a cluster of seven genes involved in the synthesis of the lipid A core of the outer membrane lipopolysaccharide that is down regulated 3-4-fold in day 4 late in cyst development (*RC1_ 0610, RC1_ 0612, RC1_0613, RC1_0615, RC1_0616, RC1_0617,* and *RC1_0618*).

Collectively, these results confirm microscopic and biochemical observations that cyst development involves significant changes in cell wall and cell membrane biochemistry that are presumably needed to allow survival in harsh environmental conditions such as extreme desiccation.

### Membrane transport

**Figure 5:**
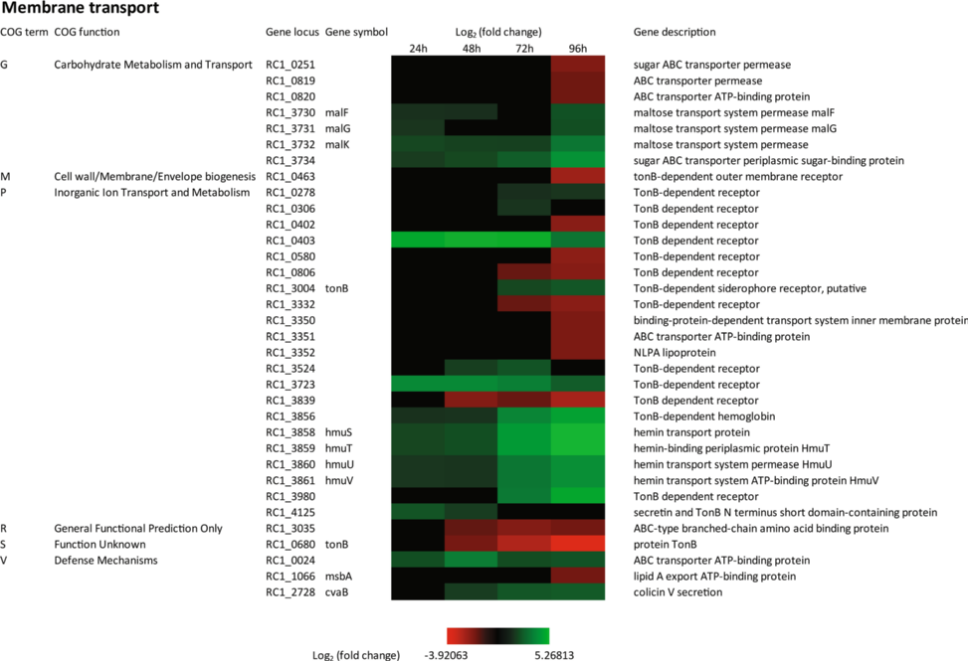
**Time course expression profiles of representative membrane transport genes.** Each column represents sample from one time-point. The maximum relative expression is green; the minimum is red.

Gram-negative bacteria typically harbor a number of TonB dependent transporters that are responsible for the transport of large molecules such as carbohydrates, vitamin B_12_ (cobalamin), ferric iron containing siderophores, etc [35-38]. The TonB system is comprised of a cytoplasmic membrane bound TonB that is complexed with two additional membrane proteins ExbB and ExbD. The TonB-ExbB-ExbD complex interacts with a variety of outer membrane bound TonB dependent receptors to facilitate transport of large molecules through the outer membrane into the periplasmic space [38,39]. Once the substrates are in the periplasm then they are transported into the cytosol via additional inner membrane transporters such as ABC transporters [40]. *R. centenum* has an operon (RC1_0680 through RC1_0683) coding for TonB, ExbB, ExbD and ExbD1 that undergoes a significant 6 to 8-fold reduction in expression late (in day 4) during cyst cell development (Figure 3). There are also six TonB dependent outer membrane receptor genes that undergo significant (typically 4-fold) reductions in expression late in cyst development (*RC1_0402, RC1_0463, RC1_0580, RC1_0806, RC1_3332,* and *RC1_3839*) and nine that undergo significant increases in expression (up to 30-fold) often early or at middle stages in cyst cell development (*RC1_0278, RC1_0306, RC1_0403, RC1_3004, RC1_3524, RC1_3723, RC1_3856, RC1_3980,* and *RC1_4125*) (Figure 5). In regards to inner membrane receptors, there are numerous genes coding for ABC transporter subunits that also undergo significant changes in expression during cyst development. This includes increased expression (up to 16-fold) of *RC1_0024, RC1_2728, RC1_3730-3732, RC1_3734,* and *RC1_3858-3861* that code for subunits of ABC transporters several of which are thought to be involved in sugar and heme transport. There are also five ABC transporter genes and operons that undergo reduction in expression (*RC1_0251, RC1_0819-0820, RC1_1066, RC1_3035, and RC1_3350-3352*). Notably among this group is RC1_1066, which is thought to be involved in export of the lipid A component of LPS and RC1_3035 which is thought to be involved in amino acid transport (Figure 5).

These alterations in transporter gene expression confirms that there are significant alterations in cell membrane composition as cells transition to cyst forms and further demonstrate that the composition of substrates that are imported/exported are also altered during cyst development.

### Carbon metabolism and energy production/storage

**Figure 6:**
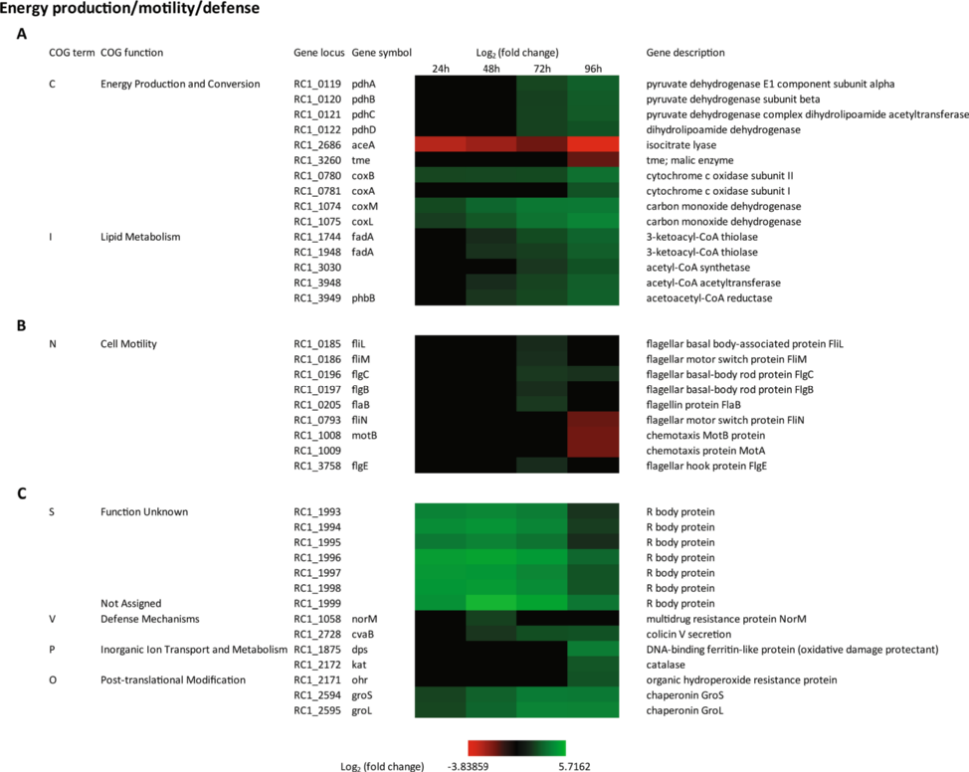
**Time course expression profiles of representative carbon metabolism and energy production/storage genes (A), cell motility genes (B) and defense genes (C).** Each column represents sample from one time-point. The maximum relative expression is green; the minimum is red.

One of the triggers for inducing cyst development is the growth on energy poor carbon sources such as the use of nutrient poor CENBA in this study [16,34]. Given that nutrient starvation is a signal for encystment it is interesting that there are no large alterations in carbon metabolism observed during cyst cell development. The few notable changes in expression include an operon coding for pyruvate dehydrogenase genes involved in the TCA cycle (*pdhA, pdhB, pdhC* and *pdhD)* that increased 6-fold in 72 hours and 96 hours of cyst development. We also found that a glyoxylate shunt pathway gene encoding isocitrate lyase *aceA* (*RC1_2686*) significantly decreased in middle and late stages of cyst developments. In addition, a gene coding for Tme that catalyzes the oxidative decarboxylation of malate to form pyruvate, is down-regulated 3-fold late in cyst development (Figure 6A).

*R. centenum* cysts are also known to contain large quantities of poly-hydroxybutyrate (PHB) [16,34]. PHB is an industrially significant biopolymer that presumably functions as an important energy storage source for cyst cells and an energy source for germination back into a vegetative state. Indeed disruption of genes for PHB production, are known to lead to a defect in formation of cysts [41]. Inspection of COG I demonstrates that there is significant increase in the expression of enzymes involved in production of poly-hydroxybutyrate (PHB) late in cyst formation. For example, five genes coding for enzymes involved in synthesis of acetoacetyl-CoA (*RC1_1744, RC1_1948, RC1_3948, RC1_3030*) and conversion of acetoacetyl-CoA to 3-OH-butyryl-CoA (*RC1_3949*) which is an immediate precursor to PHB increases 3- to 8-fold in 72 hours and 96 hours (Figure 6A). These expression results confirm biochemical data which shows that PHB production is ramped up late in cyst development.

Finally, expression of a subunit for cytochrome *c* oxidase (RC1_0780) that is involved in utilizing oxygen to form a membrane potential during respiration, is increased ~10 fold throughout encystment (Figure 6A). Oxidative stress proteins are also ramped up during this process (discussed below) so one possibility is that increased expression of cytochrome oxidase is a mechanism to reduce oxygen levels in developing cysts. There is also an increase in expression of two energy generating carbon monoxide dehydrogenases (*RC1_1074 and RC1_1075*) that convert CO and H_2_O - > CO_2_, 2H^+^ and 2e^−^ (Figure 6A).

### Cell motility

In addition to the development of cysts, *R. centenum* cells are also capable of differentiating into swim cells that have a single sheathed polar flagellum or swarm cells that have numerous unsheathed lateral flagella [34,42,43]. Swarm cell differentiation occurs when cells are grown on agar solidified growth medium or when grown at elevated temperatures (42°C-44°C) [44]. One aspect of cyst development is the ejection of flagella from vegetative cells as they round up during early stages of encystment. Accordingly, we were surprised to observe that most lateral and polar flagellar genes do not undergo significant changes in expression as cells transition to the encystment phase. The exceptions are six flagellar structural genes that have a ~3-fold increase in expression midway in cyst development (*RC1_0185, RC1_ 0186, RC1_0196, RC1_ 0197, RC1_0205,* and *RC1_3758*) (Figure 6B). In addition, the motor genes exhibit a 3-fold decrease in expression late in cyst development. These results indicate that the loss of flagella observed during cyst development may not be a result of decreased flagella gene expression but instead may be due to an as yet defined post-transcriptional event.

### Defense

R-bodies are highly insoluble protein ribbons that form distinct coiled cylindrical structures synthesized by a limited number of Gram-negative species. In several species of paramecia there are bacterial endosymbionts known as kappa particles that synthesize R-bodies which enable the host to defend themselves from predation [45-48]. Beyond kappa particles, *R. centenum,* the soil inhabiting bacteria *Pseudomonas taeniospiralis* [49,50]*, Pseudomonas EPS-5028* [51], and the free living plant pathogen *Pseudomonas avenae* [52,53] are examples of free living bacterial species that are known to synthesize R-bodies. Interestingly, the expression of an operon (*RC1_1993* through *RC1_1999*) in *R. centenum* that contains seven R-body genes is greatly induced (up to 50-fold) during cyst development (Figure 6C). Although it remains speculative, it would be intriguing if R-body formation in *R. centenum* were linked to defense of cysts against predation as it is for the kappa particle endosymbionts.

Late in *R. centenum* cyst development there is also a 5-fold increase in expression of *cvaB* which codes for an ABC type transporter of the colicin V system. In other species this transporter exports an inactive colicin V precursor that is cleaved upon transport to produce the bactericidal colicin V [54]. There is also increased expression of a *norM* gene early in cyst development, which in other species codes for a multidrug efflux pump.

Finally, there are also genes involved in defense against oxidative damage such as catalase (*kat*), *dps* and *ohr* that ramp up expression 3- to 8-fold late (96 hours) in cyst development. Expression of *groS* and *groL* that provide defense against protein mis-folding are increased 3 to 4 fold throughout cyst development (COG O) (Figure 6C).

### DNA replication

**Figure 7:**
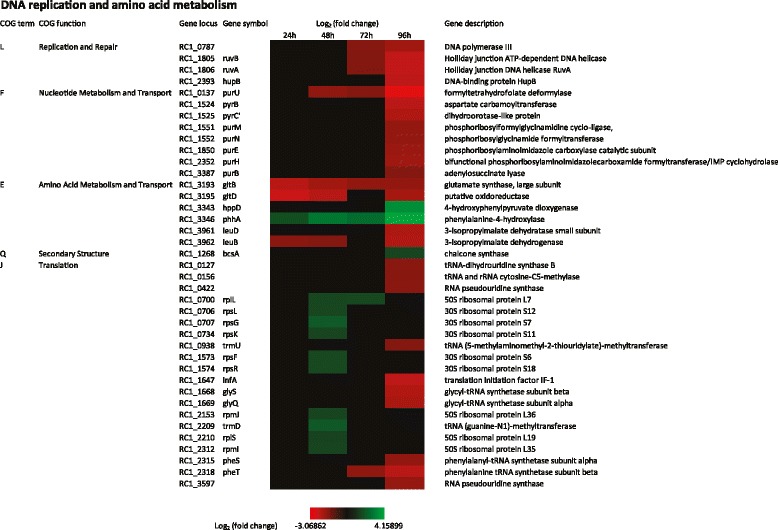
**Time course expression profiles of representative DNA replication and amino acid metabolism genes.** Each column represents sample from one time-point. The maximum relative expression is green; the minimum is red.

Given that cysts are a non-replicative dormant phase of the live cycle, it’s not surprising that there are a number of chromosome replication genes that are significantly down-regulated late (72 hours to 96 hours) in cyst development. The list includes *ruvB* and *ruvA* that code for proteins which resolve Holliday junctions and *hupB* that codes for a histone-like DNA-binding protein that is thought to stabilize DNA under extreme environmental conditions. Each of these are down regulated 4.6-fold. Expression of DNA Polymerase III is also down regulated 3.8-fold. Additionally there are six genes that code for enzymes involved in purine metabolism (*purB, purE, purH, purM, purN, and purU*) and two pyrimidine metabolism genes (*pyrB*, and *pyrC’*) that are down regulated 3.4- to 8-fold in day 4 (Figure 7). These results suggest that DNA replication is ramped down as the cells enter late stage of encystment.

### Amino acid metabolism and translation

As is the case for DNA metabolism, there is also an apparent ramp-down of protein synthesis late in cyst development. Evidence for this conclusion is centered on analysis of COG E which has numerous amino acid metabolism genes that exhibit 3 to 5-fold reduced expression late (96 hours) in cyst development. This includes genes coding for enzymes involved in lysine, valine, leucine, isoleucine aspartate, threonine, serine, glutamate, and histidine biosynthesis (Figure 7). One exception to the noted reduction in amino acid metabolism are genes coding for enzymes involved in phenylalanine biosynthesis (*hppD* and *phhA*) which exhibit a 5- to 16-fold increase in expression late in development (48 hours through 96 hours) (Figure 6). Phenylalanine is used as a precursor for flavonoid biosynthesis which also utilizes the enzyme chalcone synthase [55]. The chalcone synthase gene (RC1_1268) has previously been shown to ramp up expression late in cyst development [21]. Even though they are not well characterized in bacteria, flavonoids are involved in UV filtration in plants which is intriguing given that cyst cells also show resistance to UV killing [56].

Beyond changes in amino acid biosynthesis, cyst development also exhibits an apparent alteration in protein translation (COG J). This includes an interesting ~3-fold increase in expression of nine ribosomal protein genes at 48 hours (*rplL, rspL, rspK, rpsF, rpmJ, rplS, rpmL, rpmB, rpsG*) followed by a later 3- to 5-fold decrease in expression of four tRNA synthetase genes (*glyS, glyQ, pheS, pheT*), five tRNA modifying genes (*RC1_0127, RC1_0422, RC1_0156, RC1_3597, trmU*) and the translation initiation factor IF-1 (*infA*) (Figure 7).

### Transcription

**Figure 8:**
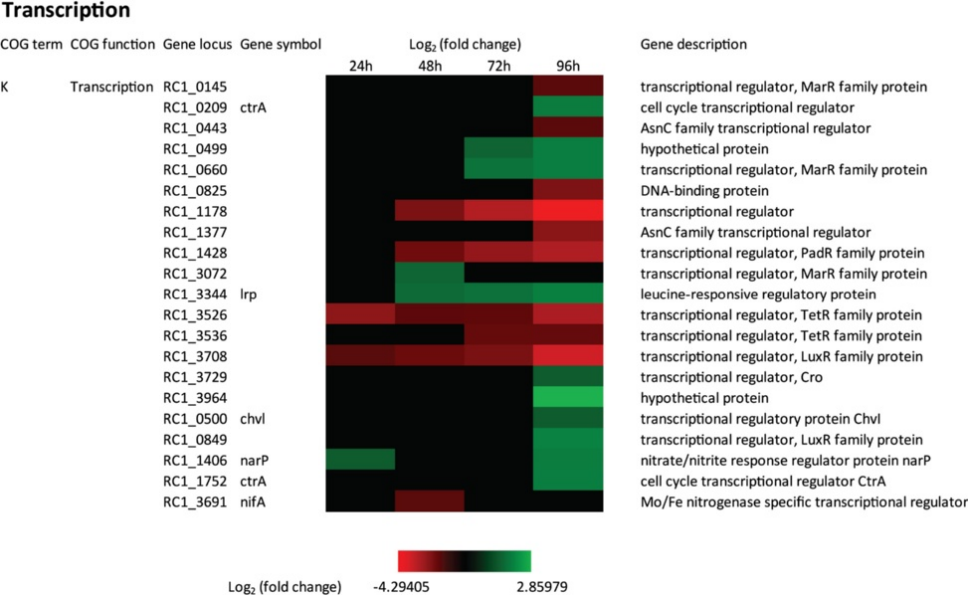
**Time course expression profiles of representative transcription genes**. Each column represents sample from one time-point. The maximum relative expression is green; the minimum is red.

A total of 71 genes in the *R. centenum* genome are annotated as transcription factors and of those, 32 are identified by this study as being differentially regulated during cyst development. Inspection of COG K shows that the expression of 20 transcription factors were up- or down-regulated by more than 3 fold. Among these, the expression of 10 members from the LysR, MarR, TetR and AsnC family of transcription factors undergo repression as the cells undergo cyst development (*RC1_0825, RC1_1178, RC1_1377, RC1_1428, RC1_3526, RC1_3708, RC1_0145, RC1_0443, RC1_3536, RC1_3691*) (Figure 8). A second set of 10 transcription factors undergo increased expression during cyst development (*RC1_0499, RC1_0660, RC1_3344, RC1_3964, RC1_0849, RC1_1752, RC1_3072, RC1_3729, RC1_0500 and RC1_1406*). A major challenge going forward is to define roles of these transcription factors in the cyst developmental process. Towards this end, several of these transcription regulators have previously been shown to play a role in the regulating cell development. For example, CtrA is a master cell cycle response regulator that contributes to swarmer cell differentiation in *Caulobactor crescentus* [57,58]. Previous research has reported that a deletion of *RC1_1752* resulted in enhanced cyst formation whereas expression of a phosphor-mimetic allele of RC1_1752 (CtrAD51E) suppressed cyst cell formation [24]. In this study, we observed that the expression of CtrA homologs coded by *RC1_0209* and *RC1_1752* are both up-regulated at 96 hours (Figure 8).

**Figure 9:**
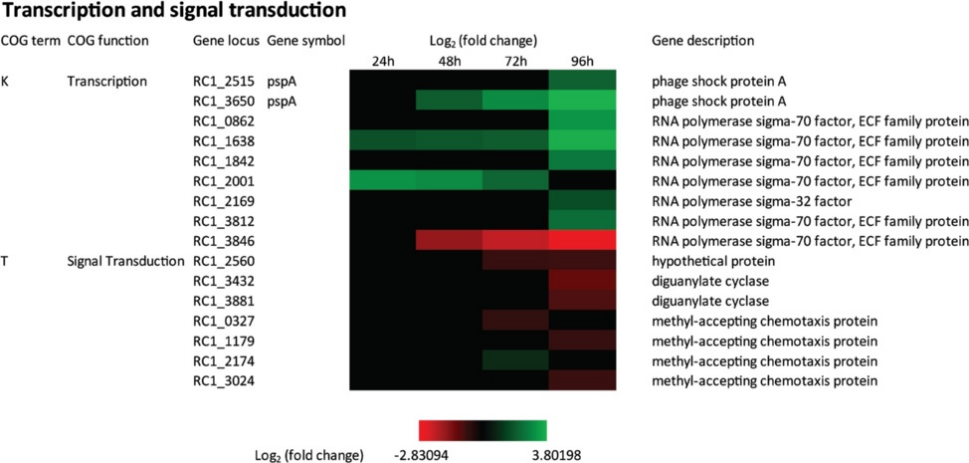
**Time course expression profiles of representative transcription and signal transduction genes.** Each column represents sample from one time-point. The maximum relative expression is green; the minimum is red.

*R. centenum* possesses 19 sigma factors that determine the promoter specificity of the RNAP holoenzyme. Altering the expression or activity of sigma factors is a mechanism commonly used by bacteria to differentially regulate gene expression of downstream pathways. Indeed, controlling expression and activity of sigma factors is a major mechanism for regulating the development of endospores in Gram-positive spore forming bacteria [59,60]. We were therefore intrigued by the observation that genes coding for seven sigma factors, six of which are in the σ70 family (*RC1_0862, RC1_1638, RC1_1842, RC1_2001, RC1_3812* and *RC1_3846*) and one in the σ32 family (*RC1_2169*), are differentially expressed during cyst development (Figure 9). Of particular interest are *RC1_1638*, *RC1_1842, RC1_3812* and *RC1_2169*. Each of these sigma factors exhibit elevated expression late in development. Indeed, we have previously reported that a mini-Tn5 disruption of a σ70 coded by *RC1_1638* leads to a defect in cyst development [27]. A second σ70 homolog coded by *RC1_2001* also shows interesting increased expression early in development while a σ70 coded by *RC1_3846* shows reduced expression late in development. Finally, there are two putative negative regulators of σ54 (*RC1_3650* and *RC1_2515*) that also undergo increased expression late in cyst cell development (Figure 9). Collectively, these results indicate that the control of cyst development does indeed involve regulating the expression and activity of alternative sigma factors.

### Signal transduction

**Figure 10:**
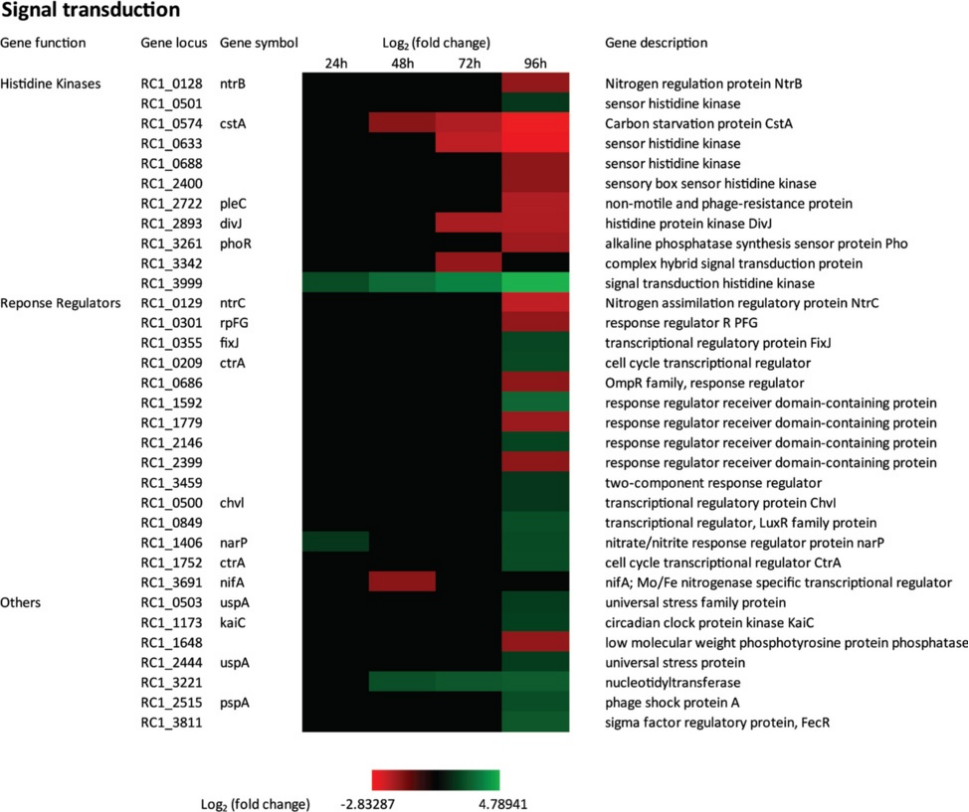
**Time course expression profiles of representative signal transduction genes.** Each column represents sample from one time-point. The maximum relative expression is green; the minimum is red.

There are 396 genes annotated as signal transduction and or regulatory in the *R. centenum* genome (9.6% of genome), of which 40 were identified as DEGs during cyst development as listed in COG T (Figure 10, Additional file 2). Analysis of our RNA-seq data indicates that numerous two-component systems (TCS), methyl-accepting chemotaxis proteins (MCPs) and secondary messengers are likely regulators of the cyst developmental process (Figure 9, Figure 10, Additional file 1).

In the *R. centenum* genome there are 55 genes predicted to encode histidine kinases, 16 of which are hybridized to receiver domains [61]. There are also 54 proteins that are predicted to contain receiver domain only or a response regulator receiver domain linked to a DNA-binding domain. Our RNA-seq data showed that there were 11 histidine kinases and 15 response regulators significantly up- or down- regulated during cyst development (Figure 10). The expression of six regulators involved in nitrogen and phosphate assimilation are altered such as *ntrB-ntrC, fixJ, nifA, phoR, narP*. With the exception of *fixJ* and *narP*, which have about 4-fold increase in expression late in development, the other nitrogen and phosphate regulators have a 3-6 fold reduction in expression late in cyst development (typically in day 4). The change in expression of numerous nitrogen regulators is notable given that cyst development can be induced during growth on rich complex medium that also contains elevated amounts of nitrogen [27].

Cell cycle and developmental regulator genes are another class that undergo changes in expression. Specifically, *divJ* and *pleC* that exhibit a ~4-fold reduction in expression late in development (Figure 10). There are two genes coding for homologs of the cell cycle regulator CtrA that show a 4-fold increase in expression late in cyst development. Mutational studies have shown that CtrA does indeed have a role in cyst development [24].

A total of 22 genes have been annotated to code for proteins harboring GGDEF, EAL and HD-GYP domains that are involved in the synthesis and degradation of cyclic di-GMP (7 with GGDEF domain, 2 with EAL domain, 7 with both GGDEF and EAL domains and 6 with HD-GYP domains) [62,63] . In this group of regulators, three diguanylate cyclases (*RC1_3432, RC1_3881, RC1_2560*) containing GGDEF domains are down-regulated 4- to 6-fold late in cyst development suggesting that cyclic di-GMP may also be an effector signal affecting cyst development (Figure 9).

## Discussion

### Encystment involves large temporal changes in metabolic gene expression

Recent genomic studies using high resolution RNA-seq technology have established that bacteria undergo large scale global reprogramming of gene expression to cope with environmental stresses. For example, recent research has shown that 1391 out of 3189 genes in *Synechocystis* sp. PCC 6803 genome are differentially expressed in response to salt stress including many genes that effect energy metabolism and protein synthesis [64]. In cases where environmental stress leads to cell differentiation, such as spore formation, large global changes in the transcriptome are known to occur as a result of complex changes in numerous regulatory networks. For example, global transcriptome analysis of myxospore formation by *Myxococcus xanthus* has revealed that a total of 1486 genes out of 6687 are differentially regulated in response to glycerol-induced sporulation [28]. A large portion involve genes in energy metabolism, protein synthesis and fate. There are also a large number of two-component regulatory systems that undergo alterations in expression pattern during myxospore formation. Lower resolution DNA array studies of *B. subtilis* during spore development also indicate that ~520 genes undergo significant temporal changes in expression [65].

**Figure 11:**
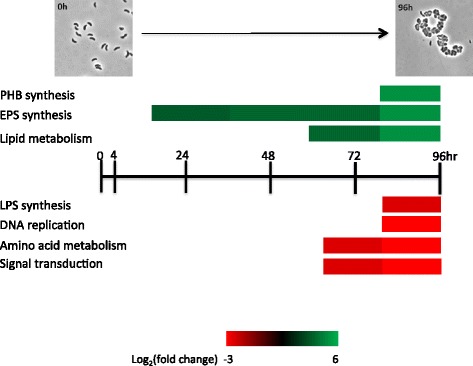
**Summary of gene expression changes observed in the CENBA culture as cyst cells develop.** Colored bars indicate average expression changes observed for the indicted cellular processes with green bars indicted in increase in expression and red bars indicating a reduction in expression.

In our study of cyst development by *R. centenum*, we have also observed the occurrence of large global changes in gene expression during cyst development. Specifically, we found that 812 out of 4105 total genes exhibit temporal changes in expression during encystment. Many observed changes could be predicted. For example, we have observed that there are large alterations in cell wall and lipid biosynthesis gene expression during cyst development (Figure 11). In *Azospirillum* studies, the cell wall of cyst cells are known to be comprised of an undifferentiated layer of polysaccharides [10,14,15]. In this study, the expression level of a number of genes predicted to be involved in cyst exine layer synthesis and transport increase over the cyst formation process. Although we have concluded that these gene expression changes are most likely a result of cyst development, it should be noted that only ~20 to 40% of the cells in the CENBA culture actually undergo cyst development. This level of development is similar to studies on *Bacillus subtilis* sporulation [7] where it has been shown that only a sub-population of sporulation induced cultures undergo development to form cysts. This we cannot rule out that some of the observed changes in expression may reflect nurse cells that do not undertake this developmental process.

In addition to changes in genes that affect cell wall/membrane composition, we have also observed changes in genes affecting carbon metabolism during cyst development. Specifically, there are numerous changes in gene expression leading to the synthesis of PHB which is known to accumulate to large levels in cyst cells (Figure 11) [10,14,16,34]. Presumably, PHB is used for energy storage for survival of dormant cyst cells. There are also alterations in the expression of enzymes that affect defense against oxidative damage.

In addition to alterations in carbon metabolism, we also observed that expression of many genes involved in the synthesis of amino acids are reduced late in cyst development (Figure 11). Cyst cells are metabolically dormant so the need for amino acid biosynthesis is likely minimal. Similarly, we have also observed reduced expression of numerous genes involved in the synthesis of nucleotides (Figure 11). There is also reduced expression of cell cycle genes such as DivJ and PleC homologs that code for proteins involved in the control of DNA replication. Again these changes in expression are not surprising as DNA replication does not occur during dormancy.

### High resolution RNA Seq analysis provides clues to regulatory cascades that control encystment

There are obvious links between cell’s capabilities to sense changes in extracellular environmental conditions with concomitant large-scale adaptation of genome expression. This process involves a combination of transporters, sensors, and phosphorylation/regulatory cascades that subsequently modulate the activity of transcription factors [66]. Our data suggests that a complex regulatory network comprised of hierarchical signal transduction pathways and transcriptional regulators are involved in controlling cyst development. Notably, we observed significant up- or down-regulation of 11 histidine kinase and 15 response regulator genes during cyst development. Indeed prior genetic studies by our laboratory have implicated the involvement of five histidine kinases in the control of cyst development [21,27]. One challenge going forward will be to determine which, if any, of the developmentally altered HK genes have direct control of cyst development or if they have indirect control. For example, many could indirectly affect cyst development by affecting metabolic pathways that when altered lead to induction of encystment.

In many studied species, altering the synthesis or activity of sigma factors can lead to changes in the expression of numerous downstream genes. Thus, temporally controlling the expression or activity of sigma factors can be a facile way of differentially programming global changes in gene expression such as what occurs during cyst development. Indeed it has been reported that bacteria with a complex lifestyle, or those that encounter diverse environmental conditions, usually possess an increased number of sigma factors [60]. In *R. centenum* there is a surprisingly large number of sigma factors (19 annotated) many of which are members of the σ70 family [61]. In regards to the role of sigma factors in dormancy, it has been shown that sporulation development in *B. subtilis* is primarily regulated by a cascade of sigma factor expression and degradation [67]. It was also reported recently that when exposed to a wide range of environmental and nutritional conditions, the transcriptome of *B. subtilis* changed drastically and 66% of the transcriptional variance occurred in regulons controlled by various RNA polymerase sigma factors [68]. Recent transcription analysis of myxospores formation by *M. xanthus* has also identified five sigma transcription factors that are significantly upregulated during glycerol induced sporulation process, of which *rpoN* is predicted to regulate spore formation and maturation [28]. Regarding *R. centenum,* the observed temporal changes in expression pattern of several sigma factor genes as identified in this study highly suggests that the cyst development process involves the use of sigma factors to control global changes in developmental gene expression. Indeed, we have observed that disruption of *RC1_1638*, which codes for a σ70, expressed late in cyst development, leads to a defect in cyst formation [27]. Clearly, additional studies on the role of the identified sigma factors is warranted and needed to understand which genes are under control of differentially expressed sigma factors and their involvement in cysts development.

## Conclusions

In extreme environmental changes such as desiccation, some species survive by forming metabolically dormant spores or cysts. How Gram-positive species form heat and desiccation resistant spores is relatively well understood. Less well characterized are mechanisms allowing Gram-negative cells to form desiccation resistant cysts. Our high resolution RNA-seq analysis provides the first detailed understanding of temporal changes in gene expression that occur during encystment in a Gram-negative organism. Multiple changes in expression occurred in genes involved in cell wall biosynthesis and cell membrane composition, which presumably allow cysts to promote cell survival in desiccating conditions (Figure 11). Genes involved in PHB energy storage are also ramped up during development. This is contrasted by reduced expression of genes involved in protein synthesis and DNA replication. The latter is not surprising as cysts are metabolically dormant and non-replicating. Numerous changes in expression of regulatory genes are also noted including several that are known to affect cyst development. Future research on these regulatory proteins will undoubtedly help our understanding of regulatory cascades that are responsible for controlling the observed changes in gene expression that occur during cyst development.

## Methods

### Bacterial strains, media, and growth conditions

The wild-type *R. centenum* strain ATCC 51521 was cultured aerobically in nutrient rich CENS medium that has pyruvate and soytone as carbon sources or in cyst inducing minimal defined CENBA medium containing 20 mM butyrate as the sole carbon source [16]. Liquid grown cells were incubated with shaking in an Erlenmeyer flask at 37°C or on grown in agar-solidified media at 42°C.

### Phase-contrast microscopy

The developmental stages of cells were monitored by phase contrast microscopy prior to harvesting for RNA extraction with an estimated level of encystment reaching ~20 to 40% at the 96 hr time point. Aliquots of cells were imaged as wet mounts on a Nikon E800 light microscope equipped with a 100x Plan Apo oil objective. Image capture was carried out with a Cascade: 1 K Megapixel EMCCD Camera and METAMORPH imaging software, v.4.5.

### RNA isolation

Wild-type *Rhodospirillum centenum* was grown aerobically overnight to stationary phase in CENS medium at 37°C. The cells were sub-cultured into CENBA medium as a 1:50 dilution inoculum. Cell morphology was monitored during a 96-hour period. Cell samples from three biological replicates at five points (4, 24, 48, 72 and 96 hours) were collected. To acquire enough total RNA from each time point, 100 ml, 50 ml, 10 ml, 10 ml and 10 ml of cell cultures were collected at 4, 24, 48, 72 and 96 hours respectively. Cell samples were homogenized using the FastPrep® Instrument (MP Biomedicals) by Lysing Matrix B in impact-resistant 2 mL tubes with RNApro™ Solution (MP Biomedicals). Total RNA samples were then extracted using FastRNA® PRO BLUE KIT (MP Biomedicals). For further cleaning-up, each total RNA sample was treated with RNeasy Mini Kit (Qiagen) following RNA clean-up protocol and eluted with 50 μl RNase-free water. TURBO DNase (Ambion) was applied to remove genomic DNA. 1.5 μl of TURBO DNase (2 Unit/ μl) and 6 μl of 10x TURBO DNase Buffer was added to 50 μl of RNA sample and incubated at 37°C for 30 min. The application and incubation of TURBO DNase was repeated once to achieve maximum DNA removal. RNA clean-up was carried out again with an RNeasy Mini Kit (Qiagen) to remove any contamination introduced by TURBO DNase buffers. Final RNA concentrations were measured by NanoDrop (Thermo Scientific). A typical OD_260_ to OD_280_ ratio of RNA samples was approximately 2.0. Further quantitation and quality control of total RNA samples were performed using a 2100 Bioanalyzer (Agilent Technologies).

### Transcript isolation, library construction and RNA-sequencing

Library construction and RNA-sequencing were conducted by University of Wisconsin-Madison Biotechnology Center DNA Sequencing Facility. Briefly, total RNA was reduced of ribosomal RNA content using an EpiCentre® Ribo-Zero™ Magnetic (Bacteria) kit with a targeted 2 μg total RNA input. Illumina mRNA-Seq libraries were prepared from rRNA-depleted samples using the TruSeq™ RNA Sample Prep kit (Illumina, San Diego, CA) per the manufacturer's protocol. Adapters containing 6 nucleotide indexes were ligated to the double-stranded cDNA and all cDNA libraries were amplified with 11 PCR cycles. Single end sequencing (1x100bp) was performed on the Illumina HiSeq 2000 according to the standard Illumina protocol. The sequences have been deposited in the National Center for Biotechnology Information’s Sequence Read Archive (accession no. SRP045612).

### Data analysis

Data analysis was carried out on GALAXY platform (http://galaxyproject.org/) [69-71]. Sequences of 100-nt in length were first trimmed using FASTQ Quality Trimmer (version 1.0.0) with a window size of 6, step size of 3 and quality score greater than 20. Trimmed sequences were mapped to the annotated 4,355,548 base pair *R. centenum* ATCC 51521 genome harboring 4,105 genes [61] using the program Bowtie (version 1.1.2). Transcripts were assembled with Reads per Kilobase of Transcripts Mapped (RPKM) values calculated using Cufflinks (v2.1.1) [72]. Mapped RNA-Seq reads were visualized using the Integrative Genomics Viewer [73]. Fold-change calculations for time course expression were undertaken pair-wised between samples of 4 hours in CENBA medium and sample at 24, 48, 72 and 96 hours in CENBA medium using Cuffdiff with geometric library normalization method and minimum alignment counts of 10 [74,75]. Genes were considered to exhibit significant differential changes in expression (DEG) when log_2_ of fold change was ≥1.5 with a false discovery rate adjusted p value of <0.05. Orthologous groups of DEGs were annotated according to eggNOG (evolutionary genealogy of genes: Non-supervised Orthologous Groups, version 4.0, http://eggnog.embl.de/version_4.0.beta/).

## Additional files

Additional file 1: Table S1. A table summarizing all of the up and down regulated genes in reach individual COG cluster. Additional file 2: A spreadsheet providing fold changes for all genes that were analyzed in this study.
