# Supplementary material for: Transcriptome analysis of cyst formation in Rhodospirillum centenum reveals large global changes in expression during cyst development
Source: BMC Genomics. 2015 Feb 13;16(1):68. doi: 10.1186/s12864-015-1250-9 (PMC4340629; doi:10.1186/s12864-015-1250-9)
Supplement: Additional file 1: Table S1. — A table summarizing all of the up and down regulated genes in reach individual COG cluster. [file 12864_2015_1250_MOESM1_ESM.docx]

**Supplemental Material**

Table S1: Differentially expressed genes grouped by COG.

| COG term | COG function | up-regulated genes | down-regulated genes | total number of genes |
| --- | --- | --- | --- | --- |
| C | Energy Production and Conversion | 25 | 15 | 40 |
| D | Cell Cycle Control and Mitosis | 0 | 2 | 2 |
| E | Amino Acid Metabolism and Transport | 10 | 25 | 35 |
| F | Nucleotide Metabolism and Transport | 3 | 12 | 15 |
| G | Carbohydrate Metabolism and Transport | 14 | 6 | 20 |
| H | Coenzyme Metabolism | 4 | 12 | 16 |
| I | Lipid Metabolism | 38 | 5 | 43 |
| J | Translation | 15 | 13 | 28 |
| K | Transcription | 19 | 13 | 32 |
| L | Replication and Repair | 2 | 13 | 15 |
| M | Cell wall/Membrane/Envelope biogenesis | 30 | 25 | 55 |
| N | Cell Motility | 6 | 3 | 9 |
| O | Post-translational Modification | 9 | 4 | 13 |
| P | Inorganic Ion Transport and Metabolism | 32 | 19 | 51 |
| Q | Secondary Structure | 17 | 2 | 19 |
| R | General Functional Prediction Only | 37 | 31 | 68 |
| S | Function Unknown | 87 | 49 | 136 |
| T | Signal Transduction | 18 | 22 | 40 |
| U | Intracellular Trafficking and Secretion | 1 | 8 | 9 |
| V | Defense Mechanisms | 3 | 3 | 6 |
|  | Not Assigned | 98 | 79 | 176 |
